# A population study on the association between leisure time physical activity and self-rated health among diabetics in Taiwan

**DOI:** 10.1186/1471-2458-10-277

**Published:** 2010-05-26

**Authors:** Chia-Lin Li, Yi-Chang Lai, Chin-Hsiao Tseng, Jen-Der Lin, Hsing-Yi Chang

**Affiliations:** 1 Department of Health Care Management, Chang Gung University, Tao-Yuan, Taiwan; 2 Department of Medical Research and Development, National Taiwan University Hospital Yun-Lin Branch and National Taiwan University College of Medicine, Taipei, Taiwan; 3 Division of Endocrinology and Metabolism, Department of Internal Medicine, Chang Gung Memorial Hospital, Chang Gung University, Tao-Yuan, Taiwan; 4 Division for Health Policy Research and Development, Institute for Population Health Sciences, National Health Research Institutes, 35 Keyan Road, Zhunan Town, Maoli County, Taiwan

## Abstract

**Background:**

There is strong evidence for the beneficial effects of physical activity in diabetes. There has been little research demonstrating a dose-response relationship between physical activity and self-rated health in diabetics. The aim of this study was to explore the dose-response association between leisure time physical activity and self-rated health among diabetics in Taiwan.

**Methods:**

Data came from the 2001 Taiwan National Health Interview Survey (NHIS). Inclusion criteria were a physician confirmed diagnosis of diabetes mellitus and age 18 years and above (n = 797). Self-rated health was assessed by the question "In general, would you say that your health is excellent, very good, good, fair, or poor?" Individuals with a self perceived health status of good, very good, or excellent were considered to have positive health status.

**Results:**

In the full model, the odds ratio (OR) for positive health was 2.51(95% CI = 1.53-4.13), 1.62(95% CI = 0.93-2.84), and 1.35(95% CI = 0.77-2.37), for those with a total weekly energy expenditure of ≥ 1000 kcal, between 500 and 999 kcal, and between 1 and 499 kcal, respectively, compared to inactive individuals. Those with duration over 10 years (OR = 0.53, 95%CI = 0.30-0.94), heart disease (OR = 0.50, 95%CI = 0.30-0.85), and dyslipidemia (OR = 0.65, 95% CI = 0.43-0.98) were less likely to have positive health than their counterparts. After stratified participants by duration, those with a duration of diabetes < 6 years, the adjusted OR for positive health was 1.95(95% CI = 1.02-3.72), 1.22(95% CI = 0.59-2.52), and 1.19(95% CI = 0.58-2.41) for those with a total weekly energy expenditure of ≥ 1000 kcal, between 500 and 999 kcal, and between 1 and 499 kcal, respectively, compared to inactive individuals. In participants with a duration of diabetes ≥ 6 years, total energy expenditure showed a gradient effect on self-perceived positive health. The adjusted OR for positive health was 3.45(95% CI = 1.53-7.79), 2.77(95% CI = 1.11-6.92), and 1.90(95% CI = 0.73-4.94) for those with a total weekly energy expenditure of ≥ 1000 kcal, between 500 and 999 kcal, and between 1 and 499 kcal, respectively, compared to inactive individuals.

**Conclusions:**

Our results highlight that regular leisure activity with an energy expenditure ≧ 500 kcal per week is associated with better self-rated health for those with longstanding diabetes.

## Background

In a summary of data collected from 1996-2005 by the Behavioral Risk Factor Surveillance System (BRFSS) [1], the Centers for Disease Control and Prevention (CDC) demonstrated that adults with diabetes were more likely to report fair or poor health than people without diabetes; a finding consistent with that of previous studies [2]. Although self-rated health is a method by which people subjectively assess their health, several studies have suggested that this simple measure is a valid measure of health status [3]. This subjective health assessment is particularly important because it has been shown to be associated with morbidity and mortality [4,5], well-being [6], and subsequent health care utilization [7,8]. In Taiwan, diabetes has been the fifth leading cause of death since 2002. However, to date no studies on self-rated health status and associated factors among Taiwanese diabetics have been published.

Most early studies on self-assessed health among the diabetic population have focused on diabetes-specific attributes, such as the duration of diabetes [9], diabetes complications [10], and the use of insulin [11]. Maddigan et al. recently used population-based data from the Canadian Community Health Survey Cycle (2000-2001) to show that the comorbidities of stroke and depression had the largest impact on health, and health behaviours such as physical activity were also an important determinant of health for people with type 2 diabetes [12]. Another recent study of a nationally representative sample of U.S. adults with diabetes aged ≧18 years demonstrated that undertaking moderate physical activity for ≧ 30 min on ≧ 5 days or vigorous physical activity for ≧ 20 min on ≧ 3 days per week was significantly associated with better self-rated general health [13]. However, for people with diabetes it remains unclear whether taking up physical activity has an independent effect on the way they assess their health, or if it acts indirectly due to the beneficial effect of physical activity on glycemic control.

Although engaging in adequate leisure time physical activity is considered to be an important self-care behavior for glycemic control among diabetics [14], the Third National Health and Nutrition Examination Survey in the US found that only 31% of adults with type 2 diabetes met the national recommendations (moderate physical activity for ≧ 30 min on ≧ 5 days or vigorous physical activity for ≧ 20 min on ≧ 3 days weekly) for physical activity, 38% reported an insufficient amount of physical activity, and another 31% reported physical inactivity [15]. Therefore, it is important to assess factors associated with poor exercise practices among diabetics that may help identify groups at high risk for physical inactivity.

Currently, the majority of studies advocate that a minimum amount of energy expenditure of 1000 kcal per week through regular physical activity is associated with health benefits [16]. However, several reports suggest that an even lower level of physical activity may be associated with health benefits in some subpopulations [16,17]. It should also be noted that the minimum amount of energy expenditure associated with health benefits may differ depending upon the health outcome of interest. In addition, the relationship between the dose of physical activity and self-rated health for people with diabetes is less clear. These observations prompted us to consider whether there was an independent dose-response relationship between physical activity and self-rated health in diabetics.

The main aim of this study was to explore the dose-response association between leisure time physical activity and self-rated health among diabetics in Taiwan after controlling for other comorbidities and diabetic related attributes. First, we investigated whether there was a dose-response relationship between leisure time physical activity and self-rated health among diabetics. Second, we examined whether leisure time physical activity had an independent association with self-rated health. Third, we examined whether the independent association between leisure time physical activity and self-rated health varied across different durations of diabetes. Finally, we identified factors associated with physical inactivity among diabetics.

## Methods

### Study population

The study population included participants in the National Health Interview Survey (NHIS) in Taiwan, 2001. This was a cross-sectional survey of a national sample based on a multistage complex design which has been described in detail previously [18,19]. The original sample consisted of the 5,798 households (22,121 subjects) who completed the survey. The response rate was 94.2% for individuals and 91.1% for households. The sampling scheme resulted in an equal probability sample with a design effect of almost 1. Therefore, the data can be treated as a simple random sample (SRS). The data is available to the public http://nhis.nhri.org.tw. There were a total of 797 individuals who had a physician confirmed diagnosis of diabetes and were aged 18 years or older. Of these 797 potential participants, we excluded 82 with missing self-rated health data, leaving 715 eligible subjects for analysis.

### Measures

Self-rated health was measured by a single question ("In general, would you say that your health is excellent, very good, good, fair, or poor?") [5]. Individuals who answered good, very good, or excellent were considered to have positive health status. Individuals were classified as inactive if they did not report engaging in any kind of leisure activity during the last two weeks. Those individuals reporting leisure time activity were asked to categorize these activities (up to three) according to a list of predefined typical leisure activities in this population. These leisure activities included walking leisurely, jogging or race walking, swimming, traditional Chinese exercise, sports, aerobics, folk dancing, bicycling, mountain climbing, weight lifting and walking up stairs. For each activity, respondents also indicated the frequency and duration (hours and minutes) of the activity during the 2 weeks prior to interview, and their perceived breathing rate (during the activity). Measures of physical activity were modified from the Nutrition and Health Survey in Taiwan [20,21]. The internal consistency of the questions for major types of exercises was 0.88. The Kappa for the reported frequency and duration of activities ranged from 0.41 to 0.46, implying acceptable validity and reproducibility. In this study, intensity refers to an assessment of the energy expenditure required to perform the activity and is expressed as a multiple of the resting metabolic rate (MET). The activity intensity for each category of activity was based on the report by Ainsworth et al. [22], as well as breathing rate during the activity [23]. The weekly energy expenditure values (kcal/week) were calculated by multiplying the corresponding MET values by frequency per week, hours spent each time, and body weight (kg), and then summing the values. This measurement of leisure time physical activity has been used to examine the relationship between components of leisure physical activity and total mortality in Taiwanese older adults [20], and to compare the prevalence of physical activity between Taiwan and the U.S. [23].

Basic demographic information and anthropometric measurements recorded in this study included age, sex, education, and body height and weight which were all obtained from the questionnaires. Body mass index (BMI) was calculated as weight (kilograms) divided by height squared (meters squared). We chose a BMI cut-off of 23 kg/m^2 ^to define overweight, based on the BMI cut-off proposed by WHO for the Asia-Pacific region [24]. Factors considered to be associated with self assessed health in diabetics were the duration of diabetes, comorbidities (such as hypertension, dyslipidemia, heart disease, or stroke), use of insulin, and other health behaviours including smoking status.

### Statistical analysis

Logistic regression analysis was performed to assess the association between self- rated positive health status and other factors. In order to compare the role of leisure time physical activity to that of other health problems and diabetic related attributes, these groups of variables were adjusted for separately and then simultaneously using multiple logistic regression. All models were adjusted for other possible confounders including age, sex, and education. Crude and adjusted odds ratios (OR) and 95% confidence intervals (CI) for self- rated positive health status were calculated. The association between physical inactivity and other factors was also examined by logistic regression. All analyses were conducted using SAS statistical software, version 9.1 (SAS Institute, Cary, NC) and SPSS statistical software, version 12.0 (SPSS, Chicago, Ill., USA).

## Results

Table 1 shows participant characteristics and their relationship with self-rated positive health status among adults with diabetes in Taiwan. Overall, 25.5% of adults aged 18 and above with diabetes rated their health as positive, and 44.6% reported no leisure activity during the last two weeks. In the crude analysis, positive health status was significantly less likely to be reported by those who were older, had a longer duration of diabetes, and had other chronic comorbidities such as hypertension, dyslipidemia, and heart disease (P-value < 0.05). Female participants, individuals using insulin and those with a history of stroke showed a trend toward being less likely to rate their health as positive (0.05 < P-value < 0.10). Positive health status was significantly associated with years of education ≧ 7 years (OR = 3.22; 95% CI = 1.96 - 5.29), reported leisure activity with a total energy expenditure of ≧ 1000 kcal per week (OR = 2.89; 95% CI = 1.87 - 4.47), and a total energy expenditure of between 500 and 999 kcal per week (OR = 1.81; 95% CI = 1.09 - 2.98).

**Table 1:** Distribution of participants' characteristics, crude Odds Ratios (OR) and 95% Confidence Intervals (CI) for positive health status among diabetics in Taiwan.

Characteristics	No. Respondents	Overall Percentage	Positive Health (%)	Crude OR (95% CI)	P-value
N	715	100	25.5		
Age (years)					
18-44	72	10.1	41.7	1	
45-64	369	51.6	26.0	0.49 (0.29-0.83)	0.008
65+	274	38.3	20.4	0.36 (0.21-0.63)	<0.001
Sex					
Male	378	52.9	28.0	1	
Female	337	47.1	22.6	0.75 (0.53-1.05)	0.093
Education* (years)					
0	161	22.6	14.9	1	
1-6	269	37.7	20.8	1.50 (0.89-2.54)	0.129
7+	283	39.7	36.0	3.22 (1.96-5.29)	<0.001
Body mass index* (kg/m^2^)					
Normal (BMI < 23)	308	54.5	26.3	1	
Overweight (BMI≧23)	257	45.5	28.8	1.13 (0.78-1.64)	0.508
Current Smoking					
No	553	77.3	24.1	1	
Yes	162	22.7	30.2	1.37 (0.93-2.02)	0.112
Duration of diabetes* (years)					
<2	183	26.1	33.3	1	
≧2 and < 6	211	30.1	27.5	0.76 (0.49-1.17)	0.208
≧6 and <10	117	16.7	23.1	0.60 (0.35-1.02)	0.058
≧10	189	27.0	19.0	0.47 (0.29-0.76)	0.002
Insulin*					
No	609	85.7	26.8	1	
Yes	102	14.3	17.6	0.59 (0.34-1.01)	0.053
Heart disease*					
No	525	75.3	29.3	1	
Yes	172	24.7	14.5	0.41(0.26-0.65)	<0.001
Hypertension*					
No	393	55.4	29.3	1	
Yes	316	44.6	20.9	0.64(0.45-0.90)	0.011
Dyslipidemia*					
No	404	61.6	29.2	1	
Yes	252	38.4	21.0	0.65(0.45-0.94)	0.021
Stroke*					
No	661	93.0	26.3	1	
Yes	50	7.0	14.0	0.46(0.20-1.03)	0.059
Total amount of energy* (kcal/week)					
inactive	308	44.6	18.5	1	
1-499	123	17.8	25.2	1.48(0.90-2.44)	0.121
500-999	110	15.9	29.1	1.81(1.09-2.98)	0.021
≧1000	149	21.6	39.6	2.89(1.87-4.47)	<0.001

*Indicates missing values.

Table 2 provides the ORs for self-rated positive health status adjusted for other comorbidities, diabetic related attributes, and leisure time physical activity. After adjustment for age, sex, and education, we found that positive health status was significantly less likely to be reported by those with a duration of diabetes of ≧ 10 years, and/or comorbidities such as heart disease and dyslipidemia (Model 1). The OR for positive health status was 2.62 (95% CI = 1.65-4.16) for those reporting leisure activity with a total energy expenditure of ≧ 1000 kcal per week, and1.77 (95% CI = 1.05-2.96) for those with a total energy expenditure of between 500 and 999 kcal per week compared to inactive individuals (Model 2). However, when the total amount of energy expenditure, other comorbidities and diabetic related attributes were included simultaneously in the same model, we found that those with an energy expenditure of ≧ 1000 kcal per week were significantly more likely to rate their health as positive (OR = 2.51; 95% CI = 1.53-4.13; P-value < 0.001), whereas those with an energy expenditure between 500 and 999 kcal per week showed a non-significant but positive trend of rating their health as positive (OR = 1.62; 95% CI = 0.93-2.84; P-value = 0.089). An energy expenditure of less than 500 kcal per week did not have a statistically significant association with self-rated health status (Model 3).

**Table 2:** Adjusted Odds Ratios (OR) and 95% Confidence Intervals (CI) for positive health status among diabetics.

	Model 1	Model 2	Model 3
Duration of diabetes (years)			
<2	1		1
≧2 and <6	0.70(0.44-1.13)		0.64(0.39-1.05)
≧6 and <10	0.60(0.34-1.08)		0.57(0.32-1.03)
≧10	0.58(0.34-0.99)		0.53(0.30-0.94)
Insulin			
No	1		1
Yes	0.83(0.46-1.51)		0.87(0.47-1.61)
Heart disease			
No	1		1
Yes	0.53(0.32-0.89)		0.50(0.30-0.85)
Hypertension			
No	1		1
Yes	0.92(0.61-1.37)		0.95(0.63-1.43)
Dyslipidemia			
No	1		1
Yes	0.66(0.44-0.99)		0.65(0.43-0.98)
Stroke			
No	1		1
Yes	0.60(0.24-1.51)		0.65(0.25-1.64)
Total amount of energy (kcal/week)			
inactive		1	1
1-499		1.37(0.82-2.30)	1.35(0.77-2.37)
500-999		1.77(1.05-2.96)	1.62(0.93-2.84)
≧1000		2.62(1.65-4.16)	2.51(1.53-4.13)

All models are adjusted for age, sex and education

The associations between leisure time physical activity and self-rated health varied across different durations of diabetes as shown in Table 3. Among diabetics with a duration of < 6 years, the adjusted OR for positive health was 1.95 (95% CI = 1.02-3.72) for those with an energy expenditure of ≧ 1000 kcal per week compared with inactive individuals. For diabetics with a duration of ≧ 6 years, the OR for positive health was 3.45 (95% CI = 1.53-7.79) for those with an energy expenditure of ≧ 1000 kcal per week, and 2.77 (95% CI = 1.11-6.92) for those with an expenditure between 500 and 999 kcal per week compared to inactive individuals.

**Table 3:** Adjusted Odds Ratios (OR) and 95% Confidence Intervals (CI) for positive health status among diabetics by different durations of diabetes.

	Duration of Diabetes < 6 years (N = 343)			Duration of Diabetes ≧ 6 years (N = 261)		
		
	**N**^**a**^	**%**^**b**^	Adjusted OR (95% CI)	**N**^**a**^	**%**^**b**^	Adjusted OR (95% CI)
Heart disease						
No	271	35.4	1	181	24.9	1
Yes	72	13.9	0.31(0.14-0.66)	80	17.5	0.80(0.38-1.69)
Hypertension						
No	206	35.9	1	135	24.4	1
Yes	137	23.4	0.84(0.49-1.44)	126	20.6	1.12(0.57-2.19)
Dyslipidemia						
No	205	34.6	1	169	24.9	1
Yes	138	25.4	0.64(0.38-1.08)	92	18.5	0.65(0.33-1.28)
Stroke						
No	329	31.6	1	232	23.7	1
Yes	14	14.3	0.54(0.11-2.71)	29	13.8	0.68(0.21-2.17)
Insulin						
No	312	31.7	1	202	24.3	1
Yes	31	22.6	0.95(0.36-2.50)	59	16.9	0.81(0.35-1.84)
Total amount of energy (kcal/week)						
inactive	152	25.7	1	105	13.3	1
1-499	61	29.5	1.19(0.58-2.41)	50	20.0	1.90(0.73-4.94)
500-999	57	29.8	1.22(0.59-2.52)	42	28.6	2.77(1.11-6.92)
≧1000	73	43.8	1.95(1.02-3.72)	64	22.6	3.45(1.53-7.79)

All models are adjusted for age, sex and education

^a ^Participants with completed data are included

^b ^Percentages of positive health

Adjusted ORs for physical inactivity in diabetics are shown in Table 4. Physical inactivity was significantly less likely to be reported by people with age ≧ 65 years (OR = 0.35; 95% CI = 0.17 - 0.69), and with years of education ≧ 7 years (OR = 0.35; 95% CI = 0.18 - 0.66). Physical inactivity was also significantly associated with use of insulin (OR = 1.87; 95% CI = 1.05 - 3.32), and being a current smoker (OR = 2.15; 95% CI = 1.33 - 3.46).

**Table 4:** Adjusted Odds Ratios (OR) and 95% Confidence Intervals (CI) for physical inactivity among diabetics (n = 504).

	**N**^**a**^	**% **^**b**^	Adjusted OR (95% CI)
Age			
18-44	61	50.8	1
45-64	273	41.0	0.54(0.29-1.00)
65+	170	31.8	0.35(0.17-0.69)
Sex			
Male	303	40.6	1
Female	201	36.8	0.87(0.55-1.38)
Education (years)			
0	66	47.0	1
1-6	190	45.3	0.77(0.42-1.40)
7+	248	32.3	0.35(0.18-0.66)
Body mass index ((kg/m^2^))			
Normal (BMI < 23)	272	40.8	1
Overweight (BMI≧23)	232	37.1	0.86(0.58-1.27)
Current Smoking			
No	375	34.1	1
Yes	129	53.5	2.15(1.33-3.46)
Duration of diabetes (years)			
<2	127	41.7	1
≧2 and <6	158	40.5	0.89(0.54-1.46)
≧6 and <10	91	39.6	0.98(0.54-1.75)
≧10	128	34.4	0.73(0.41-1.29)
Insulin			
No	433	37.4	1
Yes	71	49.3	1.87(1.05-3.32)
Heart disease			
No	386	39.4	1
Yes	118	38.1	1.01(0.63-1.62)
Hypertension			
No	284	40.8	1
Yes	220	36.8	0.91(0.60-1.39)
Dyslipidemia			
No	309	40.1	1
Yes	195	37.4	0.84(0.57-1.25)
Stroke			
No	472	39.2	1
Yes	32	37.5	0.90(0.40-2.00)

^a ^Participants with completed data are included

^b ^Percentages of physical inactivity

## Discussion

This cross-sectional study of a nationally representative sample in Taiwan shows a significant association between higher levels of leisure time physical activity and perceived positive health in diabetics. These associations remained significant even after adjusting for demographic characteristics, comorbidities, and diabetes related attributes. Furthermore, for diabetics with a ≧ 6 year duration, total energy expenditure showed a dose response relationship with self-perceived positive health.

A large body of evidence supports the beneficial effects of physical activity on diabetes management, in particular on glycemic control [14] and reduction of total and cardiovascular disease related mortality [25-27]. Few studies, however, have demonstrated a dose-response relationship between leisure time physical activity and self-rated health in a diabetic population. Our findings that diabetics achieving the recommended energy expenditure of about 1000 kcal per week through regular physical activity were more likely to report positive health are in line with another recent study [13] of a nationally representative sample of diabetics aged ≧18 years. This previous study demonstrated that moderate physical activity for ≧ 30 min on ≧ 5 days or vigorous physical activity for ≧ 20 min on ≧ 3 days per week was significantly associated with better self-rated general health [13]. Our study showed that this association between physical activity and self-rated health was independent of other comorbidities and diabetic contributors. These findings suggest that there is a direct effect of physical activity on self-rated health for diabetics achieving the recommended energy expenditure of about 1000 kcal per week. Additionally, it was evident that diabetics with a duration greater than 10 years and the two comorbidities of heart disease and dyslipidemia were significant less likely to report positive health status (Table 2, Model 1), even after adjusting for leisure time physical activity (Table 2, Model 3). These findings suggest that prevention of heart disease and dyslipidemia could be essential for improving self-perceived health among people with diabetes.

Our findings have added to the understanding of the association between leisure time physical activity levels and self-rated health among diabetics by demonstrating that reported leisure activity with an energy expenditure of more than 500 kcal per week, is positively associated with self-rated health (Table 2, Model 2). However, after controlling for comorbidities and diabetic related attributes, the association between energy expenditure between 500 and 999 kcal per week and self-rated positive health was weakened (Table 2, Model 3). This implies that for diabetics not achieving the recommended energy expenditure of around 1000 kcal per week, the potential effect of leisure activity on self-rated health is mediated by other comorbidities such as heart disease or dyslipidemia, and the duration of diabetes (Table 2, Model 3).

After controlling for comorbidities and diabetic related attributes, in diabetics with duration of illness of < 6 years, leisure activity with energy expenditure between 500 and 999 kcal per week only had a borderline significant association with positive self-rated health. However, for diabetics with duration > 6 years, leisure activity with energy expenditure between 500 and 999 kcal per week had a significant association with positive self-rated health. Previous studies have proposed that people who have had diabetes for a long time are more aware of self-care strategies in diabetes management [19], such as engaging in regular physical activity. As described theoretically by Bailis et al. [28], self-rated health may be regulated by efforts to improve one's health-related goals. In other words, respondents' self-concept may be the source of their self-rated health [28]. These observations may explain the finding that for people who have had diabetes for a long time, although the amount of exercise is less than that currently recommended, it may produce sufficient benefits on health-related goals, such as psychological and social pathways that improve individual well being and make them more likely to report positive health. These findings may have practical implications in that our data support previous suggestions that physical activity with an energy expenditure of as little as 500 kcal per week may be associated with health benefits [16,17] and that regular leisure activity with an energy expenditure of at least 500 kcal per week might be associated with improved health in terms of self-rated health. However, a prospective cohort study or an intervention study is needed to further clarify possible mechanisms.

The main limitations of this data are the potential biases introduced by the self-reported levels of physical activity, and misclassifications due to inaccurate recall. Since the study is cross-sectional in design we cannot confirm the direction of the association between physical activity and self-rated health. In addition to physical activity leading to better self-rated health, it is also possible that participants who perceive themselves to have better health are more likely to engage in physical activity, particularly in those participants with longstanding diabetes.

Despite these limitations, our study is consistent with other population-based data in regard to the low prevalence of positive self-rated health and high prevalence of physical inactivity. The present study indicates that among diabetics aged 18 and above in Taiwan, only 25.5% had positive self-perceived health, and as many as 44.6% reported no leisure activity. In the 2003 Spanish National Health Surveys, only 29.53% of adults aged 16 and above with diabetes perceived themselves to have very good or good health [29]. In the U.S. Third National Health and Nutrition Examination Survey conducted from 1991 to 1994, 42% of adults with type 2 diabetes rated their health as fair or poor and only 20% rated their health as excellent or good [30]. The Canadian Community Health Survey Cycle (2000-2001) found that 64.6% of adults aged 18 years and older with type 2 diabetes were physically inactive [12]. These results indicate the challenge involved in improving self-rated health and physical activity among people with diabetes.

Additionally, our findings emphasize the importance of developing self-perceived health promotion strategies for people with diabetes. As our results highlight the association between leisure time physical activity and self-perceived health among diabetics, self-perceived health promotion programs for diabetics should encourage increased leisure time physical activity. It has been suggested that among people with diabetes, the association between better health and healthier lifestyles, includes not smoking and higher levels of physical activity [12]. However, in our analysis, not smoking was not significantly associated with positive health. There are multiple possible reasons for this finding. One possibility is that respondents who were ever smokers would be more likely to quit smoking following worse self-perceived health. Additionally, we found that younger age, having less than a middle school education, injecting insulin, and being a current smoking were all independently associated with physical inactivity in diabetics (Table 4). This implies that smokers are less likely to have a healthy lifestyle. Our finding that participants who use insulin were more likely to report physical inactivity is consistent with previous studies [15]. Further study is needed to explore the underlying obstacles to engaging in leisure time physical activity among diabetes who use insulin. Our data suggest that health care professionals should be aware of the possible need for interventions to increase physical activity in diabetics who are younger, less educated, use insulin, or are current smokers.

## Conclusions

In summary, our results demonstrated that reported physical activity with a weekly energy expenditure of 1000 kcal was significantly associated with positive self-rated health among diabetics. A possible benefit of exercise with a weekly energy expenditure of ≧ 500 kcal was also demonstrated. These findings are particularly relevant for those with longstanding diabetes (i.e. > 6 years duration).

## Competing interests

The authors declare that they have no competing interests.

## Authors' contributions

CLL initiated the study, and drafted and revised the manuscript. YCL carried out the data analysis and participated in discussions regarding the manuscript. CHT reviewed the data and provided valuable comments on the manuscript. JDL was involved in discussions about the study and the manuscript. HYC conducted the NHIS survey, discussed the study, and reviewed and revised the manuscript. All authors read and approved the final manuscript.

## Pre-publication history

The pre-publication history for this paper can be accessed here:

http://www.biomedcentral.com/1471-2458/10/277/prepub
